# Effects of Heat Waves During Post-natal Development on Mitochondrial and Whole Body Physiology: An Experimental Study in Zebra Finches

**DOI:** 10.3389/fphys.2021.661670

**Published:** 2021-04-27

**Authors:** Riccardo Ton, Antoine Stier, Christine E. Cooper, Simon C. Griffith

**Affiliations:** ^1^Department of Biological Sciences, Macquarie University, Sydney, NSW, Australia; ^2^Department of Biology, University of Turku, Turku, Finland; ^3^Institute of Biodiversity, Animal Health and Comparative Medicine, University of Glasgow, Glasgow, United Kingdom; ^4^School of Molecular and Life Sciences, Curtin University, Perth, WA, Australia

**Keywords:** birds (Australian terrestrial), developmental plasticity, metabolic rate, mitochondria, *Taeniopygia guttata* castanotis (Aves, Passeriformes), water loss

## Abstract

Human-induced climate change is increasing the frequency, duration, and intensity of heat waves and exposure to these extreme temperatures impacts individual physiology and performance (e.g., metabolism, water balance, and growth). These traits may be susceptible to thermal conditions experienced during embryonic development, but experiments focusing on post-natal development are scant. Documented effects of heat waves on whole-body metabolism may reflect changes in mitochondrial function, but most studies do not measure physiological traits at both the cellular and whole organism levels. Here, we exposed nests of zebra finches to experimentally simulated heat waves for 18 days after hatching and measured body mass, growth rate, whole-body metabolic rate, body temperature, wet thermal conductance, evaporative water loss, and relative water economy of chicks at three ages corresponding to ectothermic (day 5), poikilothermic (day 12), and homoeothermic (day 50) stages. Additionally, we measured mitochondrial bioenergetics of blood cells 80 days post-hatch. While early-life exposure to heat wave conditions did not impact whole body metabolic and hygric physiology, body temperature was lower for birds from heated compared with control nests at both 12 and 50 days of age. There was also an effect of nest heating at the cellular level, with mitochondria from heated birds having higher endogenous and proton-leak related respiration, although oxidative phosphorylation, maximum respiratory capacity, and coupling efficiency were not impacted. Our results suggest that early-life exposure to high ambient temperature induces programming effects on cellular-level and thermal physiology that may not be apparent for whole-animal metabolism.

## Introduction

Physiological and life history traits that impact fitness can be influenced by environmental conditions, in particular temperature, experienced during early life stages (Lindström, 1999; Monaghan, 2008; Conradie et al., 2019). Exposure to high temperature can elicit stress responses (Boddicker et al., 2014), alter metabolism (O’Steen and Janzen, 1999; Moraes et al., 2003; Schnurr et al., 2014), modify water balance (Williams and Tieleman, 2000; McWhorter et al., 2018), impact growth and body size (O’Steen, 1998; Andrew et al., 2017; Andreasson et al., 2018; Sauve et al., 2021), and disrupt functional processes at the subcellular level (Paital and Chainy, 2014). Understanding the physiological impacts of high temperature is particularly important considering that anthropogenic climate change is resulting in an increase in the duration, frequency, and intensity of heat waves (Meehl and Tebaldi, 2004; Tebaldi et al., 2006; Pachauri et al., 2014; Conradie et al., 2020).

For a variety of taxa, it is hypothesized that changes in whole body metabolism and other physiological and life history traits reflect functional variation at the subcellular level, such as mitochondrial performance (Tattersall et al., 2012; Jimenez et al., 2014a,b; Hood et al., 2018a,b). Despite the crucial role of mitochondrial physiology for maintenance of homeostasis (Pörtner, 2001), few experimental studies examine temperature effects on mitochondrial function together with metabolism, growth, and body size (Price et al., 2017). Consequently, the impacts of temperature-induced variation at different levels of physiological organization are currently unclear.

Some physiological variations emerge early in life from temperature effects during the sensitive stages of embryonic development (Durant et al., 2010; Nord and Nilsson, 2011; DuRant et al., 2012; Nord and Giroud, 2020; Stier et al., 2020). Embryonic stages of all organisms are ectothermic and their mitochondria are particularly sensitive to thermal variation (Chung and Schulte, 2020). However, while phenotypic responses to high temperatures during development for some domestic animals have been investigated (e.g., Mujahid et al., 2007; Huang et al., 2015), comparatively little is known about the effects of heat during the early post-natal stage on the physiology of wild animals and potential repercussions into adulthood (Andreasson et al., 2018, 2020; Halevy, 2020).

The thermoregulatory stages of altricial birds (Price and Dzialowski, 2018) make them a useful model for examining effects of high ambient temperature (T_a_) experienced during the post-natal stage. Like embryos, nestlings are ectothermic after hatching (Price and Dzialowski, 2018), so if responses to heat observed during the egg stage are related to ectothermy, then nestlings may be as sensitive as embryos to changing T_a_ (Webb, 1987). Nestlings gradually transition to an endothermic-poikilothermic stage when their feathers begin to erupt, before finally developing full homeothermy after fledging (Sirsat et al., 2016a). Therefore, altricial passerines offer a unique opportunity for examining plastic responses to prior exposure to high T_a_ during three substantially different thermoregulatory states within the same individual.

Metabolic, hygric, growth, and mitochondrial consequences of exposure to high T_a_ may differ among populations due to genetic adaptation (Harada et al., 2019), while both acute and chronic physiological plasticity may also modify thermal responses (Williams and Tieleman, 2000; Tieleman et al., 2002, 2003; Noakes et al., 2016; Cooper et al., 2020a,b). Recent experimental data for zebra finches (*Taeniopygia guttata*) indicated that prior exposure of adults to simulated heat waves had little effect on their subsequent physiology (Cooper et al., 2020b). However, early life experiences can prepare subcellular and whole-animal phenotypes for subsequent thermal conditions (DuRant et al., 2013; Jonsson and Jonsson, 2014; Hepp et al., 2015; Gyllenhammer et al., 2020; Koch et al., 2021); these developmental effects can be as substantial as inter-population differences (Tracy and Walsberg, 2001). Physiological consequences of heat waves experienced during post-natal growth by altricial birds have received little attention, despite this being the period when thermoregulation develops. Here we examine the impact of exposure during the nestling period to simulated heat waves on physiological traits of zebra finches.

The zebra finch is an arid-habitat Australian passerine with a well-appreciated physiological capacity to withstand high T_a_ (Zann, 1996; Cooper et al., 2019). Despite this, zebra finches have been involved in mass-mortality events associated with heat waves (Finlayson, 1932; Towie, 2009). Thermal, hygric, and reproductive effects of climate change are predicted to reduce the zebra finch’s distribution and abundance (McKechnie et al., 2012; Conradie et al., 2020), but the potential for developmental plasticity to mitigate these impacts needs to be considered (Fuller et al., 2010; Boyles et al., 2011). We quantified the effects of exposure to high T_a_ (simulated heat wave) during the post-natal stage on physiological variables at the whole body and mitochondrial level. We measured metabolic rate (MR; measured as oxygen consumption, VO_2_), body temperature (T_b_), and evaporative water loss (EWL) and calculated wet thermal conductance (C_wet_) and relative water economy (RWE), at three thermoregulatory stages (ectothermic, poikilothermic, and endothermic; Sirsat et al., 2016a). We also measured the nestlings’ growth rate, and assessed mitochondrial function of red blood cells once they reached adulthood (e.g., early-life programming effects; Gyllenhammer et al., 2020).

## Materials and Methods

### Species and Housing

Groups of three male and three female zebra finches were placed in outdoor aviaries containing four shaded nest boxes. Birds were randomly selected from a population derived from wild-caught birds from western New South Wales (31.3°S, 141.6°E) and bred in captivity at Macquarie University (33.7°S, 151.1°E) for 3–7 generations. After an initial week of acclimation, the birds were provided with nesting material. Dates of clutch initiation and completion were recorded and clutches were monitored occasionally during incubation and then multiple times a day when approaching hatch date. Experiments were performed on an individual bird from each of 11 control and 11 experimentally heated nests with at least three nestlings in each nest.

### Heating Protocol

The nestling stage for zebra finches lasts 18–22 days, so nests were heated for 18 days starting on the day after hatch using a Kapton flexible heating device [Omega Engineering KHA-404(10)-P], powered by a DC regulated Powertech power supply (HW1200R-12). A Vemer digital heat regulator (HT NIPT-1P3A VM628500) set at 40 ± 0.2°C controlled the heat output *via* a digital time switch (Vemer MICRO-D) based on the nest temperature recorded by a Vemer VE122800 double injection IP68 probe placed in the nest, for 6 h a day from 9:00 until 15:00. This temperature was selected because it approaches T_b_ of small songbirds (Pollock et al., 2021) challenging their capacity for heat dissipation, and approximates the daily maximal T_a_ recorded during the breeding season within the natural habitat for this species (Griffith et al., 2016). Control nests were fitted with sham heating devices.

**Figure 1:**
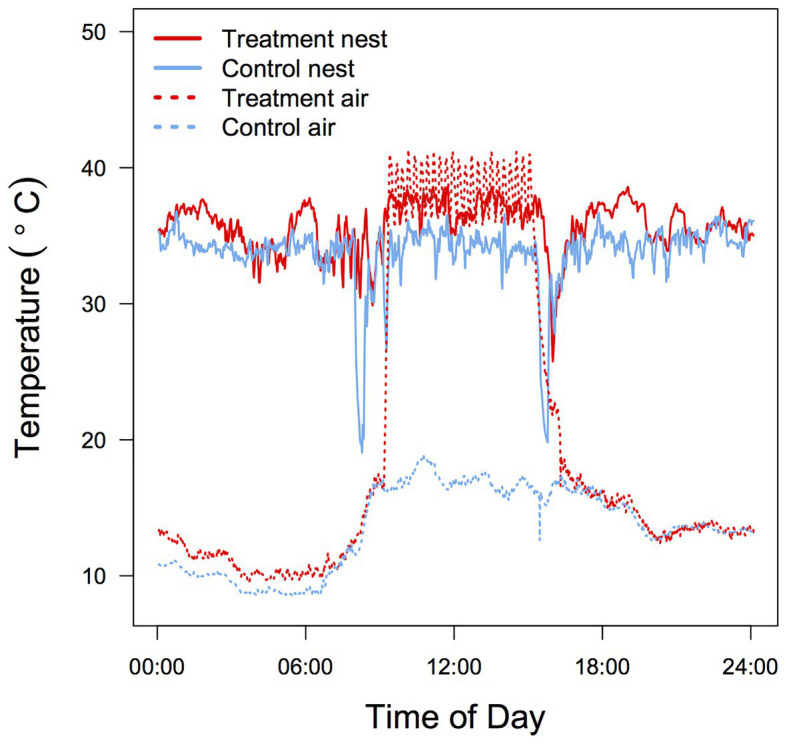
Typical 24 h air T_a_ and brood T_br_ temperature profile in control and heated zebra finch (*Taeniopygia guttata*) nest boxes. Data were recorded in adjacent aviaries during the same day for two nests with a brood size of four.

Temperature was measured every 24 s at two positions inside each nest box (3 cm below the roof = T_a_ and on the bottom of the nest in contact with the nestlings = brood temperature; T_br_) between days 1 and 8 post-hatch. Temperature was recorded only during the ectothermic period because at later stages T_br_ is influenced by the nestlings’ metabolic heat production and older nestlings tended to cluster in the corners of the box, making T_br_ data less reliable. Both temperature probes were wired to a Gemini Tinytag Plus 2 data logger and temperature data analyzed using Tinytag Explorer (ver. 4.7). During the 6 h of experimental heating mean T_a_ (37.3 ± 1.33°C) and T_br_ (35.4 ± 1.84°C) in the treatment nest boxes were significantly higher than in the control boxes (21.5 ± 1.84°C and 33.7 ± 1.33°C; *t*_1,11_ = 19.99, *p* < 0.001; *t*_1,11_ = 2.49, *p* = 0.01, respectively; Figure 1). As a consequence, the mean differential between T_br_ and T_a_ was substantially smaller for treated nests (1.9 ± 2.5°C) compared to control nests (12.2 ± 2.6°C).

### Measurements of Metabolic Rate, Evaporative Water Loss, and Body Temperature

We measured resting metabolic rate (RMR) for the second hatched nestling of each brood (and occasionally of one other sibling) at days 5, 12, and 50 post hatch, at T_a_ = 40°C, a physiologically challenging T_a_ above thermoneutrality (Calder, 1964; Cade et al., 1965; Cooper et al., 2020b). When hatching was highly synchronous, we randomly selected one individual for measurement. Measurements at day 5 were made between 15:00 and 18:00 to allow the nestling to experience regular feeding and brooding activity. At day 12 measurements commenced at 18:00 and lasted 6–9 h, until physiological variables were stable and minimal (Page et al., 2011). Prior to measurements at day 50 birds were individually caged and fasted from 14:00 (with access to *ad libitum* water); they were then measured from 19:00 until 05:00 the following morning. Nestlings aged 5 and 12 days were placed in a mesh cup within the 500 ml glass metabolic chamber, while 50 day old offspring rested on a perch.

Measurements of VO_2_, VCO_2_ and EWL were made with open-flow respirometry after Withers (2001), using a Sable Systems Foxbox analyzer. Air flow through the chamber was regulated at 100, 200, and 300 ml min^−1^ for 5, 12, and 50 day old birds, respectively, using the Foxbox’s inbuilt pump and flow regulator. Incurrent air was scrubbed of water vapor with drierite (W Hammond Co). Excurrent chamber air passed through a Vaisala 45A thin-film capacitance RH/T_a_ probe interfaced to the Foxbox, it was dried with drierite, and then passed through the Foxbox’s CO_2_ and then O_2_ sensor. The serial outputs for O_2_, CO_2_, T_a_, and RH were recorded by a PC every 20 s with a custom-written Visual Basic (VB v6) data acquisition program (P Withers). Two separate open-flow systems allowed for continuous measurement of two birds. Birds were weighed with an electronic scale (Nuweigh; ±0.01 g) before and after each measurement and the mean mass used for calculations. At the end of each experiment T_b_ (±0.1°C) was measured immediately after the bird was removed from the chamber with a plastic-tipped thermocouple (diameter 1 mm), connected to a calibrated QM1601 Digitech Thermometer (TechBrands, Australia), inserted 0.5–1.5 cm, depending on the size of the bird, into the cloaca.

Baselines of background O_2_, CO_2_, and RH were established for at least 30 min before and after each experiment. VO_2_, VCO_2_, and EWL were calculated after Withers (2001) for the ~20 min period where these values were steady and minimal, using custom-written data analysis software (VB v6; P. Withers). Since VCO_2_ mirrors VO_2_, we present only VO_2_ here. Wet thermal conductance (C_wet_, J.h^−1^.°C^−1^) was calculated as MHP/(T_b_-T_a_), where MHP is metabolic heat production, calculated from the appropriate oxy-calorific conversion (Withers et al., 2016) for VO_2_ as determined by the respiratory exchange ratio (RER). Relative water economy (RWE) was calculated as MWP/EWL, where MWP is the metabolic water production, determined from the RER and the hygric conversion for VO_2_ after Withers et al. (2016).

A Sensodyne Gillian Gilibrator was used to calibrate the Foxboxs’ flow meters. Room air (20.95% O_2_) and nitrogen (0% O_2_; BOC gases, Perth, WA, Australia) were used to two-point calibrate the O_2_ analyzers, while the CO_2_ analyzers were calibrated with a precision gas mix (0.53% CO_2_; BOC Gases) and N_2_. The RH probes were calibrated at five RHs from 2% (dry, using drierite) to 85% using a Sable Systems DG4 humidity controller.

### Growth Rate

Individual nestlings were marked with non-toxic markers on the tarsi to measure individual growth trajectories. Birds were weighed with an electronic scale (Nuweigh; ±0.01 g) and measurements were made every second day at the same time (16:00 ± 1 h) starting at hatch day until fledging (ca. day 20). The growth rate constant (*k*) was calculated using logistic regression (Ricklefs, 1968; Remeŝ and Martin, 2002; Sofaer et al., 2013) to determine the slope of the line tangent to the growth curve at the inflection point (a mass-independent estimate of growth rate), as well as asymptotic body mass (i.e., at fledging).

### Mitochondrial Bioenergetic Measurements

Avian erythrocytes are nucleated and have functional mitochondria (Stier et al., 2013), allowing measurements of mitochondrial function from small blood samples (Stier et al., 2017, 2019). Mitochondrial function of blood cells correlates with that of other tissues for birds (e.g., Stier et al., 2017) and mammals (Koch et al., 2021). Using blood rather than organs such as brain or liver allows for measurements where euthanasia is not an option, such as for repeated measurements during long-term studies, or for species of conservation concern. We, therefore, examine if high T_a_ during the post-natal stage impact blood mitochondrial function. Measurements of mitochondrial respiration were performed on intact blood cells between day 79 and 83 post hatch (hereafter referred as day 80), to prevent any impacts of sampling on the other measurements. Measurements were made for the same individuals as the other physiological variables, except in one instance when a bird did not survive until age 80; it was replaced with a sibling from the same brood. Within 5 min of capturing each bird in the aviary 70 μl of blood was taken from the brachial vein using heparinized capillaries and transferred into Eppendorf tubes. Blood cells were then immediately separated from the plasma by centrifuging the sample for 5 min at 3,000 rpm at 4°C. Plasma was removed from the upper fraction of the sample and the blood cells were then washed by adding 1 ml of ice cold phosphate buffered saline (PBS) and spinning at 800 rpm for 3 min at 4°C.

Before starting measurements of mitochondrial respiration, the PBS was discarded and the blood cells were mixed with 1 ml of MiR05 medium [0 5 mM Egtazic Acid (EGTA), 3 mM MgCl2, 60 mM K-lactobionate, 20 mM taurine, 10 mM KH2PO4, 20 mM Hepes, 110 mM sucrose, free fatty acid bovine serum albumin (1 g L^−1^), pH 7.1; Stier et al., 2017]. Blood cells were then transferred into the two chambers of an O_2_k high resolution respirometer (Oroboros Instruments, Innsbruck, Austria) set at 37.5°C for duplicate measurements for each bird. After equilibration for 10 min we recorded mitochondrial *ROUTINE* respiration, representing endogenous cellular mitochondrial O_2_ consumption. We then quantified mitochondrial O_2_ consumption associated with mitochondrial proton leak (*LEAK*) by injecting 1 μl of 5 mM oligomycin to inhibit ATP synthesis. Oxidative phosphorylation (*OXPHOS*) was calculated by subtracting *LEAK* from *ROUTINE*. We then estimated the maximum capacity of the mitochondrial electron transport system (*ETS*) by progressive titration with the mitochondrial uncoupler CCCP (carbonyl cyanide m-chlorophenyl hydrazine; 1 μl of 1 mM steps). Finally, we inhibited mitochondrial respiration by injecting 5 μl of antimycin A (a complex III inhibitor) to measure non-mitochondrial O_2_ consumption and subtract this value from each of the other parameters. We calculated two different mitochondrial flux control ratios (*FCRs*), namely an index of OXPHOS coupling efficiency [*OxCE* = 1 − (*LEAK*/*ROUTINE*)] and an index of mitochondrial reserve capacity (FCR*_R/ETS_* = *ROUTINE*/*ETS*). We evaluated the technical repeatability of mitochondrial respiration rates by calculating intra-class coefficients of correlation based on duplicate measurements (ICC, ranged from 0.63 to 0.76, all *p* < 0.001). To account for individual variation in blood cell density and differences in blood sample volume, we performed a Pierce BCA protein quantification assay (ThermoFisher Scientific, Waltham, MA, United States) and normalized mitochondrial respiration rates for the protein content of the sample by including this value as covariate in statistical analyses (see below).

### Statistical Analysis

All analyses were performed with *R* version 3.5.1 for Mac (R Core Team, 2018). We used a *t*-test to compare T_a_ and T_br_ of control and heated nests. For each nest, we estimated and compared growth rates (*K*), and asymptotic size before fledge date (*A*) for nestling mass using nonlinear mixed models (package nlme Pinheiro et al., 2017) following the methodology of Sofaer et al. (2013). Age and treatment were fixed effects, and nest and nestling identity were included as random effects to account for the lack of independence among siblings and for repeated measures of the same individual over time.

We tested effects of nest heating on whole-animal physiological variables by fitting linear mixed models using the packages lme4 and lmerTest (Bates et al., 2014; Kuznetsova et al., 2017). Treatment and age and their interaction were fixed factors and body mass was a covariate. For C_wet_, we only examined data for nestlings at 12 and 50 days because at day 5 T_b_ closely approximated T_a_, so calculation of C_wet_ was unreliable. We included random slopes for individuals nested within brood to account for repeated measures of individuals belonging to the same brood. *Post-hoc* pairwise comparisons between groups were made using the emmeans package (Lenth et al., 2019) which applies Tukey and Kenward-Roger degrees of freedom adjustments.

To examine the effect of nest heating on mitochondrial bioenergetics (*ROUTINE*, *LEAK*, *OXPHOS*, and *ETS*) and we used protein content and temperature treatment as fixed factors, and individual as a random slope to account for our repeated measurements. For analysis of *OxCE* and FCR*_R/ETS_* ratios protein content was not included as a covariate.

## Results

**Figure 2:**
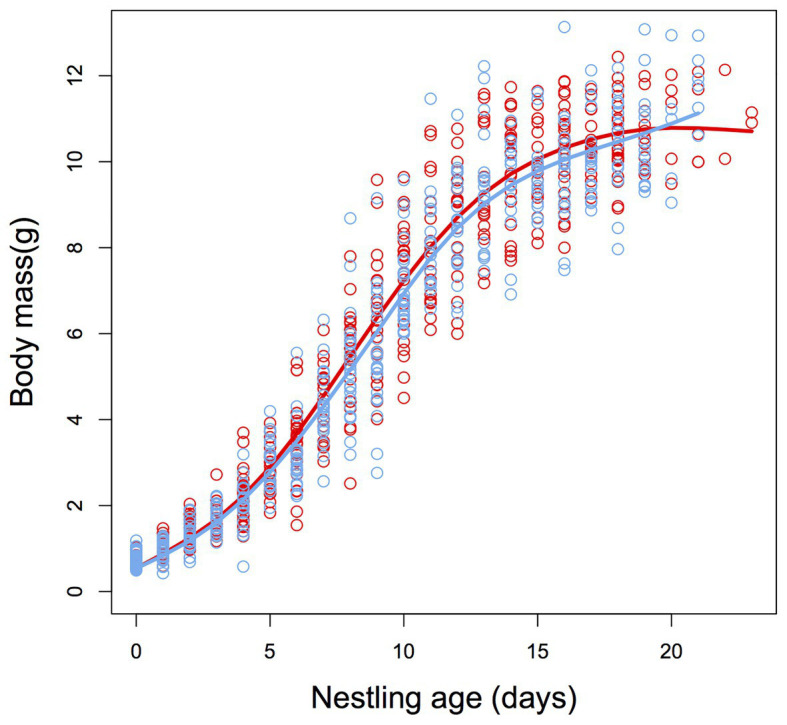
Sigmoidal plot describing the post-natal growth trajectories for body mass of zebra finch (*T. guttata*) nestlings from control (blue) and heated (red) nests. There was no significant difference in growth rate or asymptotic body mass between treatment groups (see Results section for details). *N* = 11 and 11.

Growth of nestlings did not significantly differ between control and heated nests (*F*_1,700_ = 0.33, *p* = 0.560, Figure 2), and there were no significant differences between treatment and control nests for body mass at fledging date (*F*_1,700_ = 1.53, *p* = 0.216, Figure 2) or at 50 days post-hatch (*F*_1,18.8_ = 0.08, *p* = 0.929).

**Figure 3:**
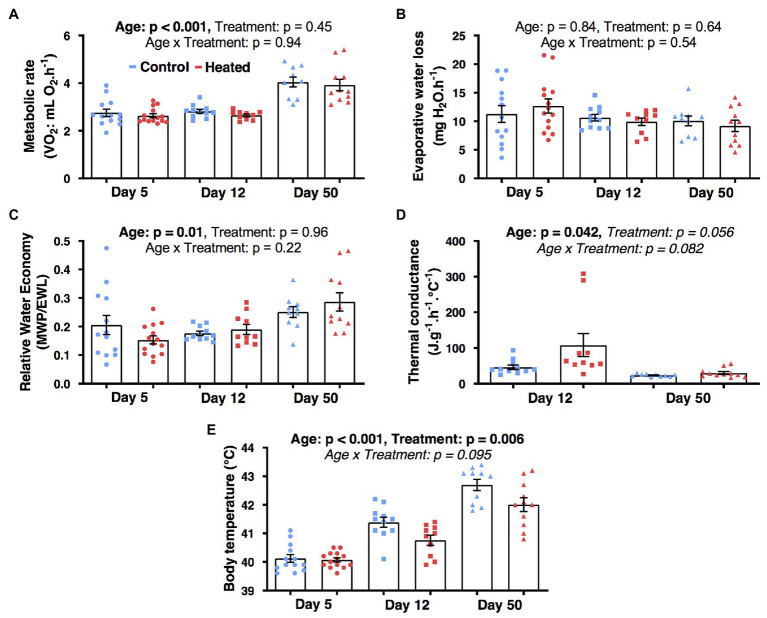
Effects of early-life heat waves exposure in zebra finch on whole-body physiology: **(A)** metabolic rate **(B)** evaporative water loss **(C)** relative water economy **(D)** thermal conductance, and **(E)** body temperature after exposure to an ambient temperature of 40°C for >2 h. Individuals have been successively measured at day 5 (ectothermic stage), day 12 (poikilothermic stage), and day 50 (homoeothermic stage; 1 month after the treatment ended) post hatch. Individual data points are presented along with their mean ± SE.

Temperature treatment had no significant influence on MR, EWL, RWE, or C_wet_ (*F*_1,62_ ≤ 4.3, *p* ≥ 0.055; Figures 3A–D). There was a significant effect of early-life heat waves on T_b_ (*F*_1,24.6_ = 9.0, *p* = 0.006; Figure 3E) with birds from heated nests having a lower T_b_ than those from control nests at day 12 (*p* = 0.026) and 50 (*p* = 0.007), but not at day 5 (*p* = 0.82). Body mass was significantly correlated with MR (*β* = 2.83 ± 0.72; *F*_1,62_ = 14.8, *p* < 0.001) and EWL (*β* = 9.54 ± 3.58; *F*_1,62_ = 7.1, *p* = 0.010), but not with the other parameters (*F*_1,62_ ≤ 1.2, *p* ≥ 0.26). Metabolic rate, T_b_, and RWE increased with age (*F*_2,62_ ≥ 4.9, *p* ≤ 0.011; Figure 3D) but C_wet_ decreased between day 12 and 50 (*F*_2,62_ ≥ 4.5, *p* < 0.042; Figure 3D) and EWL did not vary with age (*F*_2,52.8_ = 0.2, *p* = 0.83; Figure 3B).

**Figure 4:**
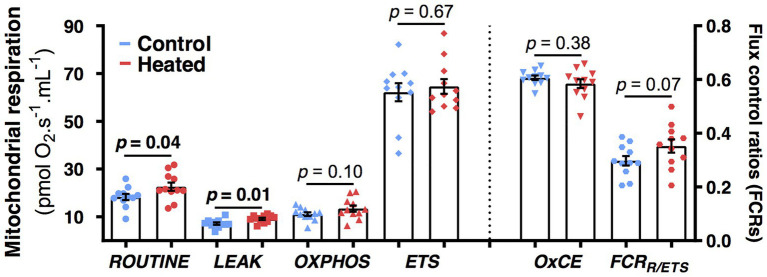
Effects of early-life heat waves exposure in zebra finch on blood cells mitochondrial physiology measured at day 80 (i.e., 2 months after the treatment ended). *ROUTINE* represents the endogenous mitochondrial respiration, *LEAK* the proton-leak related mitochondrial respiration, *OXPHOS* the ATP-synthesis related mitochondrial respiration, and *ETS* the maximal mitochondrial respiration. OXPHOS coupling efficiency (*OxCE*) is an index of mitochondrial efficiency to produce ATP and FCR*_R/ETS_* an index of the mitochondrial reserve capacity. Individual data points are presented along with their mean ± SE and describe oxygen consumption not corrected by protein content (see Materials and Methods and Results sections for details on statistics).

Birds from heated nests had higher mitochondrial respiration rates at day 80 than those from control nests for both *ROUTINE* (*F*_1,19.6_ = 4.6, *p* = 0.04; Figure 4) and *LEAK* (*F*_1,20.0_ = 8.0, *p* = 0.01; Figure 4). There was no significant effect of the heat treatment for *OXPHOS*, *ETS*, *OxCE*, or *FCR_ROUTINE/ETS_* (*F*_1,19–20_ ≤ 3.5, *p* ≥ 0.07; Figure 4).

## Discussion

For zebra finches, early-life exposure to simulated heat waves had no significant impact on whole-body metabolic rate or hygric physiology but reduced T_b_ of nestlings at days 12 and 50 (i.e., both during the temperature treatment and 1 month after it ended). A lower T_b_ allows greater scope for hyperthermia at high T_a_ (Tieleman and Williams, 1999), but can impose limits on muscle functionality and immune reactions (Boyles et al., 2011). Despite no whole-body metabolic effect, birds from heated nests had higher mitochondrial respiration (both endogenous and proton-leak) than birds from control nests 2 months after the heat treatment. While the consequences of these mitochondrial changes for accommodating high T_a_ at later life stages are unknown, our results indicate that post-natal developmental programing of mitochondrial function by exposure to elevated T_a_ does occur. Our results also suggest that the effects of early-life heat exposure emerge after the ectothermic stage (day 5) when endothermy develops. This information improves our understanding of species responses and potential resilience to climate change (Conradie et al., 2020).

Previous thermal acclimation and acclimatization studies for adult birds, including zebra finches, document lower MR and EWL, and higher T_b_, for birds during or immediately following or chronic or acute periods of exposure to high T_a_ (Williams and Tieleman, 2000; Cooper et al., 2020a). These responses create a more favorable trade-off between hyperthermia and dehydration for birds exposed to high T_a_. However, our findings are consistent with those for adult zebra finches (Cooper et al., 2020b), that suggest that prior acute exposure to high T_a_ has little effect on whole-animal metabolism and water balance at later time-points. As these physiological variables correlate with field energy and water expenditure (Nagy, 1987; Cooper et al., 2003), prior experience of high T_a_ is also unlikely to influence daily energy and water requirements in the field. So while there is evidence that birds adjust their physiology during periods high T_a_, our results support the hypothesis that periodic extreme events such as heat waves do little to prepare birds for future warming, and are of greater concern for the persistence of avian populations (McKechnie and Wolf, 2010; Franklin et al., 2014; Cooper et al., 2020a,b).

We detected no effect of early-life exposure to increased T_a_ on growth rate and body mass at fledging or at 50 days of age, consistent with observations of Hsu et al. (2020) for wild pied flycatchers (*Ficedula hypoleuca*). Dawson et al. (2005) also found that growth rates of tree swallows (*Tachycineta bicolor*) from heated nests were the same as those from control nests, although birds from heated nests had higher body mass. However, other studies report lower growth rates for nestlings from heated nests (Rodríguez and Barba, 2016; Andreasson et al., 2018). Various factors, such as physiological and parental care variables, can influence the relationship between nest temperature and nestling growth rate. In colder climates, warmer minimum and mean nest temperatures are correlated with higher nestling growth rates, while in warmer climates, higher nest temperature can negatively impact nesting growth rates (Larson et al., 2015) presumably as a consequence of nest temperature moving closer to or further from an optimal temperature for growth. The absence of a temperature effect on body mass differs from previous results for wild and captive zebra finches (Andrew et al., 2017, 2018). However, thermal responses can vary depending on the ontogenetic stage (Halevy, 2020) and in these previous studies, heating occurred during the embryonic as well as the post-natal period.

There is a considerable body of literature discussing the impact on physiological and life history variables of manipulating temperature during the embryonic stage (Elphick and Shine, 1998; O’Steen, 1998; Gilchrist and Huey, 2001; Du and Ji, 2003; Ardia et al., 2010; Nord and Nilsson, 2011; DuRant et al., 2012; Scott and Johnston, 2012; Hepp et al., 2015; Andrew et al., 2017; Ton and Martin, 2017) in contrast to the scarcity of manipulative studies during the post-natal stage for altricial birds (Andreasson et al., 2020). The significant later-life whole body physiological consequences of high T_a_ during embryonic development compared to our observations for nestlings suggests that the embryonic phase may be more sensitive to plastic adjustments compared to the post-natal stage. This may be because embryos of all species are ectothermic with potentially greater exposure to variable temperature. However, our experimental treatment induced no whole-animal metabolic or water loss effects, even for ectothermic 5-day-old nestlings. This, together with the absence of T_a_ effects for body mass and growth rates compared to previous findings (Andrew et al., 2017, 2018) support the idea that conditions experienced during the post-natal stage have fewer subsequent effects for adult birds than those experienced as an embryo. This may be a consequence of the developmental and gene expression processes underlying functional differentiation of tissues during the embryonic stage (Gilbert and Epel, 2009), while the post-natal stage is characterized by cellular proliferation and growth rather than functional differentiation (Starck and Ricklefs, 1998).

We did uncover some functional consequences of early-life exposure to heat waves at the subcellular level. Two months after the end of the temperature treatment, both endogenous (*ROUTINE*) and proton-leak (LEAK) mitochondrial respiration rates were higher for birds in experimentally heated nests compared to control nests. It is possible that this is a consequence of birds from control and heated groups differing with respect to the thermal optimum for their mitochondrial function. High proton LEAK is functionally linked to thermogenesis in brown adipose tissue of mammals and has been associated with a fast pace of life and higher oxidative stress for birds (Jimenez et al., 2014b). A small increase in proton leak reduces reactive oxygen species (ROS) production and oxidative stress (Divakaruni and Brand, 2011; Koch et al., 2021). Acute exposure to heat generates cellular stress and increases production of ROS (Abele et al., 2002; Mujahid et al., 2005; Tan et al., 2010), which can elicit an upregulation of mitochondrial proton leak as a protective mechanism (Jarmuszkiewicz et al., 2015). Consequently, the increase we observed in LEAK for birds from heated nests may be advantageous for buffering heat-induced oxidative stress. However, higher LEAK increases metabolic heat production, which is detrimental at high T_a_ and it is unclear why our birds from heated nests had lower rather than higher T_b_ despite the elevated mitochondrial function. Future studies should examine this by measuring long-term implications for T_b_, oxidative stress, and biomarkers of biological aging, such as telomere length (Dupoué et al., 2017).

A correlation between whole-animal metabolism and mitochondrial function is expected (White and Kearney, 2011; Jimenez et al., 2014a), but the interrelationships between physiological function at different levels of organization are debated (Norin and Metcalfe, 2019). The increased mitochondrial ROUTINE and LEAK, we observed at 80 days post-hatch for our heat-treated birds did not reflect an increased whole-body metabolism at early life-history stages and T_b_ was lower, not higher. This may be due to temporal physiological changes (we observed changes in rates of whole-body metabolism at different ages) and/or elevated C_wet_ for birds from heated nests which may have dissipated increased MHP. Our results suggest that elevated C_wet_ may have occurred but we unfortunately did not have sufficient statistical power to detect differences in C_wet_ for heated and control nets (Figure 3D). There may also be differences in mitochondrial contribution to whole-animal metabolism from different tissues (Else and Hulbert, 1985). Mitochondrial VO_2_ of muscle or liver, which have a greater contribution to whole-animal metabolism than blood (Sirsat et al., 2016b,c), may have correlated more strongly with whole-body metabolism, but this is unlikely as mitochondrial function of blood correlates with that of other tissues (Stier et al., 2017). These findings emphasize the need for improved understanding of the relationship between cellular and whole body metabolism, and of the utility of blood as a tissue for mitochondrial measurements (Koch et al., 2021).

In summary, our study provides no evidence that exposure to high nest temperature during the neonatal period has any subsequent effect on growth rate, mass, and whole-body metabolic and hygric physiology of zebra finch chicks during any of the three phases of thermoregulatory development. These results may reflect the natural history of the species (Griffith et al., 2021). Yet, there is an impact on T_b_ and on ROUTINE and LEAK mitochondrial function for endothermic birds, consistent with responses to oxidative damage and life-history tradeoffs (Tan et al., 2010; Hood et al., 2018a,b). This suggests that there is some limited scope for prior experience of heat waves during the neo-natal phase to result in whole organism metabolic changes. Such results force us to reconsider our approaches in exploring environmental influences on physiological traits. Indeed, failing to detect metabolic or growth consequences at the whole body level does not exclude sub-cellular repercussions that may have important implications for other unmeasured aspects of phenotypic and evolutionary fitness.

## Data Availability Statement

The data used to produce the results reported in the paper are publicly available at: https://osf.io/s4hyq/files/.

## Ethics Statement

The animal study was reviewed and approved by the Macquarie University and Curtin University Animal Ethics Committees (ARA 2017/024 and ARE2017-16).

## Author Contributions

RT, CC, and SG designed the study and provided resources and logistical support. RT conducted the experiments and statistically analyzed the resulting data. CC and AS contributed to data collection and analyses. RT, AS, CC, and SG wrote the manuscript. All authors contributed to the article and approved the submitted version.

### Conflict of Interest

The authors declare that the research was conducted in the absence of any commercial or financial relationships that could be construed as a potential conflict of interest.
